# Value of observational studies in the outcome evaluation of transcatheter and surgical aortic valve replacement

**DOI:** 10.1093/icvts/ivae198

**Published:** 2024-11-28

**Authors:** Fausto Biancari, Timo Mäkikallio, Paola D’Errigo, Gianluca Polvani

**Affiliations:** Department of Cardiovascular Surgery, Centro Cardiologico Monzino, IRCCS, Milan, Italy; Department of Medicine, South-Karelia Central Hospital, University of Helsinki, Lappeenranta, Finland; National Center Global Health, Istituto Superiore di Sanitá, Rome, Italy; Department of Cardiovascular Surgery, Centro Cardiologico Monzino, IRCCS, Milan, Italy; Department of Biomedical, Surgical and Dental Sciences, University of Milan, Milan, Italy

**Keywords:** Aortic valve stenosis, Aortic stenosis, Transcatheter, Aortic valve replacement, Low-risk

Transcatheter aortic valve replacement (TAVR) has the merit of having extended the invasive treatment of aortic valve stenosis (AS) to high-risk patients. The minimally invasive nature of TAVR is an attractive and effective alternative treatment method to surgical aortic valve replacement (SAVR) also in lower-risk patients as substantiated by the results of recent randomized clinical trials (RCTs) [1, 2]. This has slowly resulted in a widespread use of TAVR in younger patients with lower operative risk and in the fading role of the Heart Team in the decision-making process. Therefore, TAVR is currently performed in patients without severe frailty and significant comorbidities and long-life expectancy. However, a recent pooled analysis of studies, including low-risk patients who underwent TAVR or SAVR, demonstrated similar midterm survival in RCTs, while observational studies reported significantly better survival after SAVR compared to TAVR [3]. In these observational studies, SAVR was associated with a survival benefit over TAVR, which became significant 2 years after the procedure. A similar, non-significant trend towards better survival after SAVR was also observed in the PARTNER 3 trial [2].

RCTs are at the top of the hierarchy of evidence in clinical medicine [4]. High-quality observational studies can provide risk estimates comparable to RCTs [5]. Still, the results of observational studies can be affected by biases related to patient selection and unmeasured confounding factors, which cannot be addressed by current methods of risk adjustment. However, in some instances, the efficacy of a treatment method has been unequivocally demonstrated in observational studies without the need to perform a RCT [6]. Furthermore, observational studies can provide evidence of how the results of RCTs translate into clinical practice [4] and evaluate the consistency of clinical outcomes of RCTs when these are potentially weakened by methodological biases [7]. Data from observational studies can even rival those from RCTs [8]. Overall, RCTs and observational studies can provide important information in the interpretation of the safety, efficacy and durability of treatment methods in patients populations with different characteristics [9].

In this issue of the Journal, Krasniqi *et al*. [10] reported the results of a multicentre retrospective study including patients who underwent isolated SAVR or isolated transfemoral TAVR at 4 Danish hospitals. The authors compared the results of these treatment methods adjusting the late adverse events for baseline characteristics using a propensity score matching analysis, which yielded 468 pairs of patients with well-balanced covariates between the study cohorts. Noteworthy, the mean age of the matched cohorts was 75 years, and their median EuroSCORE II was 1.5%, suggesting a low-risk profile and a relatively long-life expectancy of the matched study population. Thirty-day mortality after TAVR and SAVR were 0.6% and 1.3%, respectively (*P* = 0.32). Kaplan–Meier estimate of 5-year all-cause mortality was 29.8% after TAVR and 16.9% after SAVR (HR 2.05, 95% CI 1.55–2.71) at 5 years. Furthermore, SAVR was associated with significantly lower rates of cardiovascular death, sudden cardiac death, as well as lower cumulative incidences of stroke/transient ischaemic attack and permanent pacemaker implantation at 5 years. SAVR was associated with increased risk of postoperative atrial fibrillation compared to TAVR. Cumulative incidences of reoperation on the aortic valve prosthesis were comparable in the study cohorts. Subgroups analysis showed comparable late outcomes after TAVR and SAVR in patients with a EuroSCORE II 4–8%, left ventricular ejection fraction 30–50% or <30%, diabetes mellitus and active smokers. Consonant adjusted risk estimates for the late adverse events were observed in the entire study population.

The retrospective nature is the major methodological limitation of this study. Furthermore, the authors did not provide data on the presence of coronary artery disease and patients’ frailty. However, it is still unclear whether coronary artery disease may have a negative prognostic impact after aortic valve replacement [11]. We may expect that the prevalence of severe frailty might have been non-significant in most of low-risk patients, but the lack of this information is a major limitation of this analysis. The authors did not report data on early adverse events, which is an important piece of information of these patients, which could be crucial in understanding why certain patients may fare better with one approach over the other.

Despite these limitations, this study has several strengths, which deserve to be considered. First, data were retrieved from 2 national registries, which guaranteed the reliability of long-term outcomes, and this represents the major strength of the analysis. Second, the sample size of the propensity score-matched series seems to be large enough. A *post-hoc* sample size analysis based on an estimated hazard ratio of 2.05 (alpha 0.05, beta 0.80) showed that 68 patients per cohort would have been enough to reject the null hypothesis. Third, all SAVR patients included in this analysis received a Perimount bioprosthesis. This bioprosthesis has shown excellent durability among the elderly and might have mitigated the bias of using bioprostheses with suboptimal durability as implanted in previous RCTs [1]. Finally, all procedures were performed in public hospitals without reimbursement restrictions, and the authors did not have any conflict of interest related to the content of this study.

This study did not provide evidence of any causative factor leading to suboptimal late outcomes after TAVR compared to SAVR, but it confirmed the results of other observational studies pooled in a recent meta-analysis by Sá *et al.* [3]. Taken together, these findings suggest that further high-quality studies are needed to get conclusive results on the benefits and harms of performing TAVR or SAVR in different subsets of patients with AS. It is of overwhelming importance to gather data on patient’s frailty, socioeconomic status and other confounding clinical variables not included in current risk scoring methods, which might have prognostic significance in patients with AS. Meanwhile, a judicious multidisciplinary decision-making process in the management of patients with AS is recommended.

**Conflict of interest:** None declared.
